# Routine PSA Testing: An Analysis of the Controversy Concerning Its Use

**DOI:** 10.1100/tsw.2005.21

**Published:** 2005-02-16

**Authors:** Namath S. Hussain

**Affiliations:** Johns Hopkins University School of Medicine, 1620 McElderry Street, Suite #7A3, Baltimore, MD 21205, USA

**Keywords:** PSA, prostate cancer, controversy

## Abstract

Prostate cancer is the leading cause of cancer in American males today. PSA screening has been used for over 10 years as an important diagnostic tool for the disease. Because of its lack of sensitivity and specificity, however, PSA testing should be used with caution.

Prostate cancer is the most commonly diagnosed cancer and the second leading cause of death from cancer in men in the U.S. Every year, 37,000 men die from this disease[1]. The enormous public health implications of reducing morbidity and mortality from prostate cancer have lent themselves to a serious and often intense debate over screening measures.

Since the early 1990s, more and more physicians in the U.S. have come to adopt a policy of screening for prostate cancer in men over the age of 50 using a blood test for prostate-specific antigen (PSA). Although randomized controlled clinical trials are underway (the Prostate, Lung, Colorectal, and Ovarian Cancer Screening Trial) to make an evidence-based claim on the topic, currently there are no high-quality scientific data supporting the use of this screening measure. An analysis of the arguments for and against PSA testing for prostate cancer screening in the current literature will evince data concerning the established cost and harm of routine testing and the lack of data showing benefits of testing[2].

PSA is an enzyme produced by prostate epithelial cells. Its normal endogenous function is to cleave seminogelin proteins in semen. Some of the enzyme leaks out of prostatic ducts and into the bloodstream. Serum PSA levels are commonly elevated in prostate cancer, which makes them convenient markers for the cancer. A PSA blood level of above 4.0 ng/ml is usually considered a positive result and indicates that further workup is necessary, including possibly biopsy and invasive surgery. However, it is important to note that many prostate tissue–confined tumors produce PSA at levels that overlap with levels found in men without prostate cancer[3]. So, how do we differentiate? Is it really a good test? This is where the uncertainty begins. More uncertainty regarding the efficacy of the PSA test will come to the forefront as we consider arguments for and against screening efforts utilizing PSA.

PSA testing, when evaluated as a widespread public health screening measure, has a very low degree of sensitivity and specificity. Almost 20% of prostate cancers are found in men with normal PSA levels

(<4.0 ng/ml). These tumors are often completely enclosed by normal prostate tissue. In addition, only about 25% of men who have positive PSA test results (>4.0 ng/ml) will have prostate cancer detected on subsequent biopsy, which is the gold standard in prostate cancer diagnosis[1]. This positive predictive value (PPV) of a positive PSA test predicting prostate cancer falls even lower in younger age groups. For men under 50 years of age with a PSA level of 4.1–6 ng/ml, the PPV is only 12%. This means that only 12% of men with a positive PSA test will actually be found to have prostate cancer[1]. Thus, the biological test itself is neither a sensitive nor a specific indicator of whether someone has prostate cancer. Continuing to use it in screening programs would be ineffective due to the large number of false negatives (underdiagnosing men with prostate cancer) and false positives (overdiagnosing men without prostate cancer). These two undesirable consequences of the test can be attributable to the fact that PSA is not a great marker for prostate cancer and that we should begin exploring other screening possibilities rather than continuing to use this faulty test.

Perhaps the most important harm of routine PSA testing is overdiagnosis. False-positive results on the screening test can expose the patient to subsequent invasive testing and aggressive therapies with significant morbidity, such as radical prostatectomy. The false-positive results place an unnecessary and distressing emotional burden on patients and their families. Presenting a patient with the possibility of a fatal cancer is an enormous emotional burden that not only is psychologically difficult to handle, but may also lead to medical sequelae that often result from increased levels of anxiety and stress, like gastrointestinal ulcers, nausea, immune compromise, and irritable bowel symptoms. Health-care providers should take into account these potential harmful consequences of PSA testing before making a decision about whether or not to recommend screening.

Etzioni et al. studied the phenomenon of overdiagnosis through an analysis of the National Cancer Institute's SEER (Surveillance, Epidemiology, and End Results) database and computer modeling. They found that the overdiagnosis rates were 29% for whites and 44% for blacks[4]. Thus, between 29 and 44% of men diagnosed with prostate cancer after PSA screening would unnecessarily be subject to the significant morbidity of often aggressive treatment. This study's methods reveal the degree of conservatism with which the investigators approached the subject. They defined overdiagnosed patients as patients who were detected by PSA screening, but did not survive long enough to have a cancer clinically diagnosed. Most conventional studies define it as those patients who were detected by PSA screening, but did not survive long enough to develop the signs and symptoms of cancer. Because prostate cancer is a relatively indolent cancer, the signs and symptoms develop fairly late, usually many years after the clinical diagnosis is made, which allows for more time for a competing cause to kill the patient before the prostate cancer does, which would inflate the number of overdiagnosed patients[5]. Thus, even though the investigators approached the subject from a conservative point of view, giving PSA testing the benefit of the doubt, they still found significant levels of overdiagnosis. In addition, the investigators came to the conclusion that the majority of screen-detected cancers would have presented clinically and only a minority of the prostate cancers found at autopsy would have been detected by PSA testing[4], revealing the lack of necessity or benefit of having the test in place as a public health measure. Other analyses of data from the SEER database have revealed that there has been a stage migration of diagnosis, yielding a lower mean age of treated patients. Although younger patients are being treated for prostate cancer and mortality rates have dropped since 1991, these data suggest that this drop in mortality is probably not due to screening. In order to be sure of a cause-and-effect relationship between screening and reductions in mortality, we must analyze the results of randomized trials examining the effects of screening, something that cannot be done until all of the data from the PLCO trials are fully analyzed. There has been one trial that purported to show a decrease in mortality as a result of screening by Labrie et al., however, there are several flaws in the way data were both collected and analyzed by these investigators[6]. This study followed 46,486 men, aged 45–80, who were randomized in 1988 to either screening or no screening. A PSA level of 3.0 ng/ml was used as the upper limit of normal. Among the 14,231 unscreened controls, 74 deaths occurred while 10 deaths occurred among the 7,348 screened men during the first 11 years following randomization. Median follow-up time was 7.93 years. There was a 62% reduction in cause-specific mortality in the screened men ($p = 0.005$).

An idea that has been suggested to increase the sensitivity and specificity of PSA testing is to use levels of PSA derivatives along with serum PSA levels. Using these data in concert has been shown in some studies to increase the diagnostic accuracy of serum screening for prostate cancer[7,8,9].

Apart from the psychologically mediated harmful effects of overdiagnosis, there exists significant harm in the subsequent workup and therapy that a patient might be exposed to unnecessarily. Most people sign up for free public PSA testing or get tested in their doctor's office because most of the health information available to the public touts the benefits of PSA screening and actively promotes it. Knowledge of the limitations of PSA testing in terms of the lack of sensitivity and specificity is low[10,11]. Although the data supporting PSA testing are weak, most people believe it to be an effective test and are unaware of the controversy surrounding it. Even life insurance companies are beginning to require PSA testing before the issuance of policies[12]. Overdiagnosis can lead to significant morbidity and mortality with unnecessary treatment being administered whether it be surgery, radiation, or hormonal therapy. After suspicion is aroused by elevated PSA levels, subsequent biopsies and prostatectomies can lead to erectile dysfunction, incontinence, internal bleeding, hematuria, infection, adhesions, and even death from anesthesia[2].

Because most patients are unaware of the untoward consequences of prostate cancer therapy and because they are being barraged with media flaunting the benefits of PSA testing, there exists little room for a negative feedback mechanism by which a patient can be unsatisfied with his care and then complain. This presents a significant public policy problem. There is no incentive or reinforcement that accompanies a recommendation *against* PSA screening. Regardless of the test result, the physician suggesting the test is positively "reinforced", meaning that he receives feedback from the patient that he should continue practicing medicine in the same manner. If the test is negative, the patient is reassured and thanks the physician for comprehensive care. If the test is positive, the patient is grateful to the physician for detecting the problem early (he assumes that there is a problem). Even if aggressive therapy is subsequently pursued, an impotent or incontinent patient will still reinforce the physician positively by attributing his survival to early detection and surgery. The chances are also slim that the physician will get any negative feedback along the lines of the fact that sometimes early detection and aggressive treatment do not work[13].

Some investigators have employed literature reviews to gather data about the changes in the prevalence of prostate cancer and have tried to correlate that information with PSA screening. However, Coldman et al. studied the relationship between PSA screening and subsequent changes in mortality from prostate cancer. In the study, there was no association found between PSA screening intensity and decreases in prostate cancer mortality[14].

It is important to keep in mind that even if PSA testing correctly diagnoses prostate cancer, we are still not out of the woods. People may still be exposed to aggressive treatment unnecessarily because there are a significant number of prostate cancers that do not kill patients. More men die with prostate cancer than as a result of it[15]. On the other hand, there are a sizeable number of patients whose cancer has progressed to such a stage that they cannot be helped. In these cases, PSA testing does not decrease mortality or increase life expectancy in any way.

The controversy surrounding PSA testing for prostate cancer screening will undoubtedly rage on until randomized clinical trial data become available. Until then, we must look at the data we have and make rational decisions based on limited information. Because we know that there are many significant harms, downsides, and limitations to PSA testing and we are unaware of many benefits to the screening measure, we must choose to quit promoting a flawed health policy. If we are all anxiously waiting for the coveted "evidence-based" data from the PSA randomized clinical trials that are ongoing, why are we now currently ignoring the *evidence* we have before us?
